# Supplementary material for: Saccharomyces boulardii (CNCM I-745) improves intestinal damage in sepsis by remodeling intestinal flora structure
Source: Front Cell Infect Microbiol. 2025 Jul 31;15:1602792. doi: 10.3389/fcimb.2025.1602792 (PMC12350329; doi:10.3389/fcimb.2025.1602792)
Supplement: Supplementary file 1 [file DataSheet1.zip › summary/summary/7_Differential_analysis/1_diff_abundance/3_Class/Group.DW0_vs_CW0_vs_BW0_vs_AW0/Group.DW0_vs_CW0_vs_BW0_vs_AW0_diff_barplot.pdf]

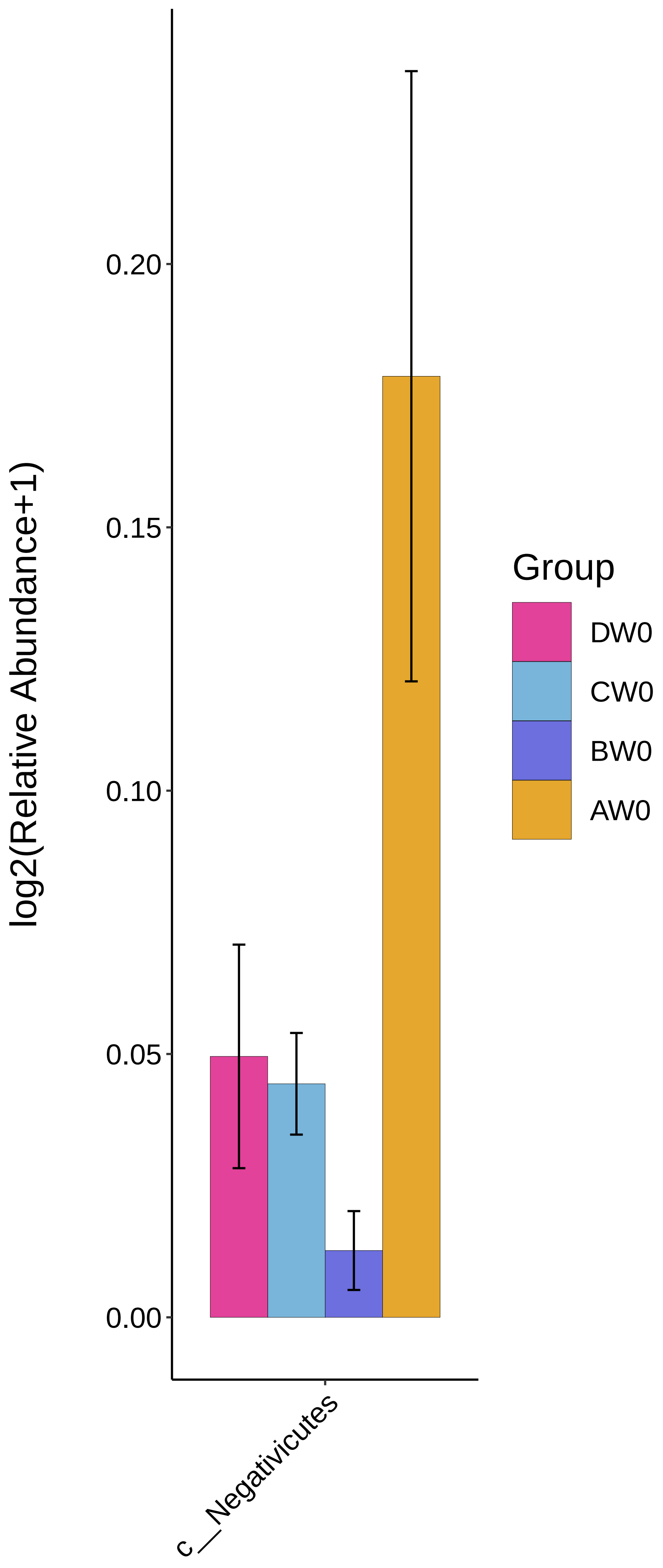

log2(Relative Abundance+1)
0.20
0.15
0.10
0.05
0.00
c__Negativicutes
Group
DW0
CW0
BW0
AW0
